# Acute Social Defeat Stress Increases Sleep in Mice

**DOI:** 10.3389/fnins.2019.00322

**Published:** 2019-04-03

**Authors:** Shinya Fujii, Mahesh K. Kaushik, Xuzhao Zhou, Mustafa Korkutata, Michael Lazarus

**Affiliations:** ^1^International Institute for Integrative Sleep Medicine (WPI-IIIS), University of Tsukuba, Tsukuba, Japan; ^2^Ph.D. Program in Human Biology, School of Integrative and Global Majors, University of Tsukuba, Tsukuba, Japan

**Keywords:** social defeat stress, slow-wave sleep, homeostatic sleep need, mouse model, slow-wave activity

## Abstract

Social conflict is a major source of stress in humans. Animals also experience social conflicts and cope with them by stress responses that facilitate arousal and activate sympathetic and neuroendocrine systems. The effect of acute social defeat (SoD) stress on the sleep/wake behavior of mice has been reported in several models based on a resident-intruder paradigm. However, the post-SoD stress sleep/wake effects vary between the studies and the contribution of specific effects in response to SoD or non-specific effects of the SoD procedure (e.g., sleep deprivation) is not well established. In this study, we established a mouse model of acute SoD stress based on strong aggressive mouse behavior toward unfamiliar intruders. In our model, we prevented severe attacks of resident mice on submissive intruder mice to minimize behavioral variations during SoD. In response to SoD, slow-wave sleep (SWS) strongly increased during 9 h. Although some sleep changes after SoD stress can be attributed to non-specific effects of the SoD procedure, most of the SWS increase is likely a specific response to SoD. Slow-wave activity was only enhanced for a short period after SoD and dissipated long before the SWS returned to baseline. Moreover, SoD evoked a strong corticosterone response that may indicate a high stress level in the intruder mice after SoD. Our SoD model may be useful for studying the mechanisms and functions of sleep in response to social stress.

## Introduction

All living organisms respond to any external biological source of stress, i.e., perceived or actual threats, with a predictable biological pattern in an attempt to restore the internal homeostasis of the body (Chrousos, 2009). Acute stress typically involves transient responses of biological systems; however, prolonged activation of these initially beneficial reactions by excessive or chronically repeated stressors may lead to stress-related disorders. Social conflicts are a major source of stress for humans and play a major role in the pathogenesis of affective disorders like anxiety and depression (Charney and Manji, 2004; Huhman, 2006). Social conflicts also occur in animals (Toyoda, 2017). A social animal with the inability to dominate its opponent shows submissive behavior and accepts a lower social rank to avoid injury and death that could occur if the animal continues to act in an aggressive manner (Huhman, 2006). Rodent models of social defeat (SoD) stress are often based on a resident-intruder paradigm where the resident attempts to defend its home cage from the intruder who eventually subordinates itself to the unfamiliar territorial resident conspecific. Acute SoD stress in rats has been shown to increase the heart rate and body temperature by sympathetic stimulation and blood corticosterone levels by activation of the hypothalamic–pituitary–adrenal axis (Koolhaas et al., 1997; Kataoka et al., 2014). These stress responses quickly dissipate and return to baseline levels within hours after the end of the stress period. SoD stress, however, also induces longer-lasting behavioral and physiologic changes. For example, several days may be required to normalize the circadian rhythmicity of body temperature, open-field behavior, and body mass growth after acute SoD (Meerlo et al., 1996a,b,c). Moreover, social animals are known to develop depression-like behaviors in response to chronically repeated SoD stress (Von Frijtag et al., 2000; van Kampen et al., 2002; Rygula et al., 2005; Huhman, 2006). For example, chronic SoD stress in mice induces anhedonia, anxiety, and social avoidance (Golden et al., 2011; Henriques-Alves and Queiroz, 2015). The anxiety and social avoidance behaviors in mice after chronic SoD stress can last several weeks (Berton et al., 2006; Tsankova et al., 2006; Krishnan et al., 2007). These long-lasting behavioral changes in animals that experience repeated SoD stress are commonly associated with alterations in gene expression patterns in the brain (Tsankova et al., 2007).

The sleep/wake cycle is regulated by multiple factors, including homeostasis, circadian rhythm, and external environment, that are integrated by specific neuronal circuits controlling the sleep/wake behavior (Weber and Dan, 2016; Eban-Rothschild et al., 2017). Stress promotes wakefulness and inhibits sleep during the period when enhanced arousal is necessary to cope with external challenges. Alterations in the sleep architecture are often observed after an acute stress exposure (Sanford et al., 2015). A sleep rebound may be induced in animals to compensate for the sleep loss during stressful situations, but it is believed that sleep alterations are not only a homeostatic response to the sleep loss but also created by the stress. The type of sleep that is induced and the extent to which sleep stages are enhanced are highly variable between the types of stress or even the study design. For example, acute immobilization or restraint stress is followed by a selective increase in rapid eye movement sleep (REMS) (Rampin et al., 1991; Meerlo et al., 2001). Moreover, mice experiencing escapable foot shock stress showed an increase in REMS, whereas mice treated with inescapable foot shock stress showed a decrease in REMS (Sanford et al., 2010). Sleep is considered to have an important role in coping with stressful situations (Goldstein and Walker, 2014).

The acute effect of SoD stress on sleep has been reported in several rodent models. Although slow-wave activity (SWA) is consistently enhanced during sleep after SoD stress in mice and rats (Meerlo et al., 1997; Meerlo and Turek, 2001; Kamphuis et al., 2015; Henderson et al., 2017), the extent to which sleep stages are promoted varies between SoD studies and protocols. Male C57BL/6j mice when defeated by aggressive mice of the same strain during a 1-h interaction period showed a strong increase of slow-wave sleep (SWS) (Meerlo and Turek, 2001), whereas only a small increase of the SWS amount was observed in another study using the same SoD procedure (Vaanholt et al., 2003). This difference may be explained by variations in the aggressive behavior of the resident mice or prior stress experiences of the intruder mice in the laboratory environment. When highly aggressive male CD-1 mice were used in another study for the SoD of C57BL/6j mice during a 5-min interaction period followed by a 20-min period of olfactory, visual and auditory contact between the resident and intruder mice, a SWS increase in the intruder mice was preceded by an increase of wakefulness (Henderson et al., 2017). A fixed interaction period likely leads to painful attacks of the CD-1 mouse on the intruder mouse and thus, the sleep-wake behavior of the intruder mice may also be affected by pain. Overall, the extent to which the SoD procedure (e.g., pain or sleep deprivation) contributes to the post-SoD stress sleep/wake effects is not well established.

In the present study, we developed a mouse model of SoD stress based on a resident-intruder paradigm to evoke sleep alterations in the intruder C57BL/6j mouse after acute SoD stress by the resident CD-1 mouse trained to display persistent aggression against the intruder mouse. To prevent painful attacks to the submissive intruder mouse, the intruder and resident mice were separated by a partition when the intruder mouse showed clear submissive behavior and was attacked by the resident mouse. We found that SWS strongly increased in response to SoD, whereas REMS was only moderately increased for a limited period several hours after the SoD session.

## Materials and Methods

### Animals

Male C57BL/6j mice (13–20 weeks old, and weighing 26–33 g), maintained at the International Institute of Integrative Sleep Medicine of the University of Tsukuba, were used in the experiments. Male CD-1 (retired breeders) mice were obtained from Japan SLC (Hamamatsu, Japan). The animals were housed in an insulated and soundproof recording chamber maintained at an ambient temperature of 23 ± 0.5°C with a relative humidity of 50 ± 5% and an automatically controlled 12 h light/12 h dark cycle (illumination intensity ≈ 100 lux). All animals had free access to food and water. This study was performed in strict accordance with the Guide for the Care and Use of Laboratory Animals of the US National Institutes of Health (2011). Experimental protocols were in compliance with relevant Japanese and institutional laws and guidelines, and approved by the University of Tsukuba animal ethics committee (protocol #14-322). Every effort was made to minimize the number of animals used as well as any pain and discomfort experienced by the animals.

### Stereotaxic Surgery for Placement of the EEG/EMG Electrodes

Mice were anesthetized with pentobarbital (50 mg/kg, intraperitoneal [i.p.]) and then placed in a stereotaxic apparatus. Electroencephalogram (EEG) and electromyogram (EMG) electrodes for polysomnographic recordings were chronically implanted in the mice (Oishi et al., 2016). The implant comprised two stainless steel screws (1 mm in diameter) that served as the EEG electrodes inserted through the skull above the cortex (anteroposterior, +1.0 mm; left-right, -1.5 mm from bregma or lambda) according to the atlas of Paxinos and Franklin (2004). Two insulated, stainless steel Teflon-coated wires, serving as the EMG electrodes, were placed bilaterally into both trapezius muscles. All electrodes were attached to a micro connector and fixed to the skull with dental cement.

### Stress Procedures

The SoD stress protocol was designed based on the resident-intruder paradigm. To prepare aggressive resident mice, male CD-1 mice (body weight > 40 g) were singly housed and trained to display persistent aggression against male C57BL/6j mice by placing a male C57BL/6j mouse in the cage of a CD-1 mouse one or two times a week. During the training, the C57BL/6j mouse was immediately removed from the cage when attacked and defeated by the CD-1 mouse. Unsuitable CD-1 mice that showed little aggression or extreme violent behavior (i.e., mice that inflict serious injuries to their opponent) were excluded from the study. Only CD-1 mice showing aggression against C57BL/6j mice within 1 min were used in the experiments to ensure successful SoD. The C57BL/6j mice used for aggression training were not used for any SoD experiments.

**FIGURE 1:**
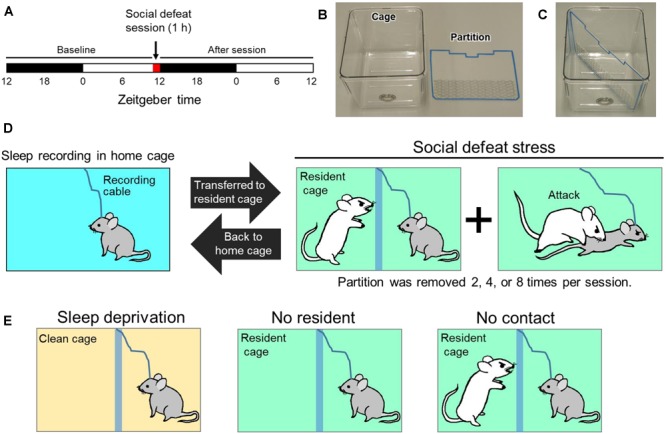
Protocol for social defeat (SoD) stress based on a resident-intruder paradigm. **(A)** Schedule of sleep recording and SoD session. **(B)** Cage used for SoD or EEG/EMG recording and partition for diagonal separation of the cage. **(C)** Cage with inserted partition. **(D)** Schematic diagram of the SoD procedure. The intruder mouse was placed behind the partition of the resident cage. The partition was repeatedly removed for multiple defeat experiences of the intruder mouse by the resident mouse during a 1-h period. After the SoD session, the intruder mouse was returned to the home cage for sleep recording. **(E)** Control experiments to differentiate between the specific effects of SoD and non-specific effects of the SoD procedure. The mouse was placed in the cage for 1 h under each condition.

For SoD stress, we used a transparent acrylic partition placed diagonally in a rectangular cage to separate the intruder and resident mice. The partition has a wire-mesh opening in the lower part for olfactory, visual, and auditory contact (Figure 1B,C). A SoD session lasted 60 min starting at zeitgeber time 11. The C57BL/6j mouse (intruder mouse) was placed behind the partition in the resident mouse’s cage. After 5 min, the partition was removed and re-inserted when the intruder mouse was attacked by the resident mouse and showed clear submissive behavior, including submissive posture, escaping, or freezing behavior. During one SoD session, the removal of the partition was repeated two, four, or eight times at 25-, 15-, or 7-min intervals, respectively. After the SoD session, the intruder mouse was returned to its home cage (Figure 1D).

The following experiments were also conducted to differentiate the specific effects of SoD and non-specific effects of the SoD procedure (Figure 1E): (1) C57BL/6j mice were placed in an unused cage with bedding, food pellets, and a partition for 60 min and sleep deprived by cage tapping (“Sleep deprivation” session). (2) C57BL/6j mice were placed in a cage with a partition previously used by a CD-1 mouse for more than 5 days (“No resident” session). (3) The intruder mouse was placed in the resident cage while the intruder and resident mice were separated by the partition for 60 min (“No contact” session). Sleep deprivation by cage tapping was not conducted during the “No resident” and “No contact” sessions.

### Vigilance State Assessment Based on EEG/EMG Polygraphic Recordings

One week after surgery, the mice were individually housed in cages in an insulated soundproof recording chamber and connected to the EEG-EMG recording cables for 2–3 days of habituation before starting the polygraphic recordings. Before the SoD session, some mice were subjected to a “Sleep deprivation” or “No resident” session, followed by a “No contact” session. Sessions were run every 3 or 4 days. Baseline was recorded on the day prior to each session (Figure 1A). The EEG/EMG signals were amplified, filtered (EEG 0.5–30 Hz; EMG 20–200 Hz), digitized at a sampling rate of 128 Hz, and recorded using the data acquisition software SleepSign^®^ (Kissei Comtec, Matsumoto, Japan). The vigilance states were classified offline in 10-s epochs of three stages, i.e., wakefulness, REMS, and SWS by SleepSign^®^ (ver 3.4) according to standard criteria (Oishi et al., 2016). As a final step, the software-defined vigilance stage of each 10-s epoch was visually examined, and manually corrected when necessary. Spectral analysis of EEG by fast Fourier transform was performed, and the EEG power densities were calculated in the range of 0.5–25 Hz in 0.5-Hz bins. SWA during SWS was calculated based on EEG power in the range of 0.5–4 Hz. In a 3-hourly plot of SWA, the data were presented as percentages of the mean of the 12-h baseline SWS during the dark period.

### Behavioral Analysis

Animal behaviors during “No resident” and “No contact” sessions were analyzed using video recordings. Vigilance states and behaviors during wakefulness were scored in 4-s epochs. Behaviors were scored as grooming, exploration (including ambulation, rearing, digging, and sniffing), consumption (eating and drinking), or quiet waking, when the behavior accounted for more than 50% of the epoch.

### Blood Sampling and Corticosterone Measurement

Blood sampling was performed by cardiac puncture with the mice under deep isoflurane anesthesia immediately after the SoD sessions with 2, 4, or 8 defeats. Blood samples were obtained within 2 min, which is rapid enough to ensure that the stress imposed by the blood-sampling procedure did not affect the corticosterone levels in the plasma (Riley, 1960). For the control group, mouse blood was collected immediately after the “No resident” session. For basal corticosterone levels, blood was collected from undisturbed mice in their home cages at zeitgeber time 12. All the mice were used only once and not subjected to any behavioral tests. Blood samples were collected into EDTA-coated syringes and immediately centrifuged at 10,000 rpm for 15 min at 4°C. Plasma samples were collected and stored at -80°C until the assay was performed. Plasma corticosterone was measured in duplicate wells using the DetectX^®^ ELISA kit (Arbor Assays, Ann Arbor, MI, United States). The mean of intra-assay coefficients of variations (*n* = 31) calculated from duplicate wells was 2.82%.

### Statistical Analysis

**FIGURE 2:**
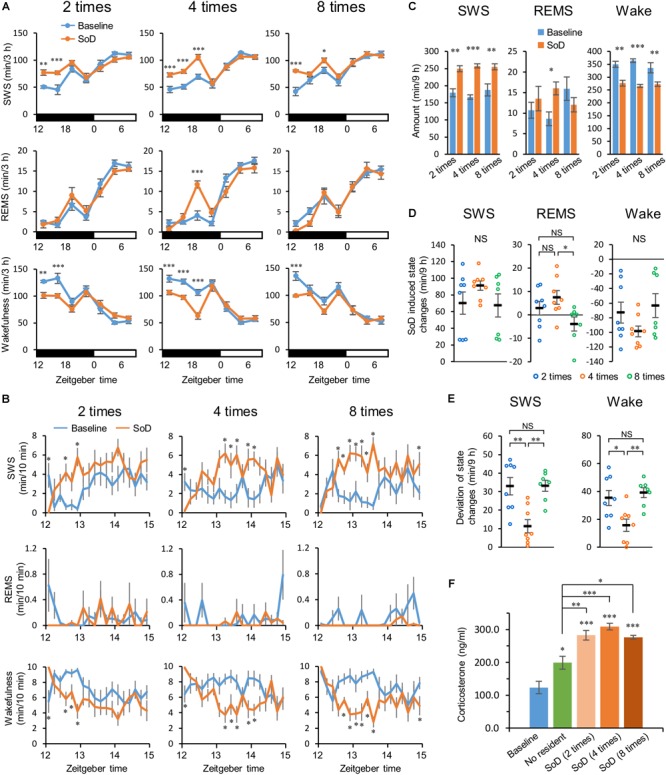
Slow-wave sleep (SWS) increased after social defeat (SoD) stress. **(A)** Time-course of SWS, rapid eye movement sleep (REMS), and wakefulness for 21 h after SoD sessions with two (*n* = 8), four (*n* = 8), or eight (*n* = 7) defeats. ^∗^*P* < 0.05, ^∗∗^*P* < 0.01, and ^∗∗∗^*P* < 0.001 compared with baseline, assessed by two-way repeated measures ANOVA followed by Bonferroni’s *post hoc* comparisons. **(B)** Time-course of SWS, REMS, and wakefulness for 3 h after SoD sessions. ^∗^*P* < 0.05 compared with baseline, assessed by paired two-tailed Student’s *t*-test. **(C)** Total amounts of SWS, REMS, and wakefulness for 9 h after SoD sessions. ^∗^*P* < 0.05, ^∗∗^*P* < 0.01, and ^∗∗∗^*P* < 0.001 compared with baseline, assessed by paired two-tailed Student’s *t*-test. **(D)** Changes in total amounts of SWS, REMS, and wakefulness for 9 h between baseline and after SoD sessions. ^∗^*P* < 0.05, compared between groups, assessed by the Kruskal–Wallis test (SWS and wakefulness) or one-way ANOVA (REMS) followed by Bonferroni’s *post hoc* comparisons. **(E)** Deviation of changes in total amounts of SWS and wakefulness. The deviation was calculated by subtracting the mean from the experimental values of each animal for changes in the total amounts of SWS and wakefulness for 9 h in each condition. ^∗^*P* < 0.05 and ^∗∗^*P* < 0.01, compared between groups, assessed by one-way ANOVA followed by Bonferroni’s *post hoc* comparisons. **(F)** Blood plasma corticosterone levels from undisturbed mice (*n* = 6) at zeitgeber time 12 of the baseline day or mice after a “No resident” (*n* = 7) or SoD sessions (*n* = 6). ^∗^*P* < 0.05, ^∗∗^*P* < 0.01, and ^∗∗∗^*P* < 0.001 compared between groups, assessed by one-way ANOVA followed by Bonferroni’s *post hoc* comparisons. Data are presented as means ± SEM. NS, not significant.

**FIGURE 3:**
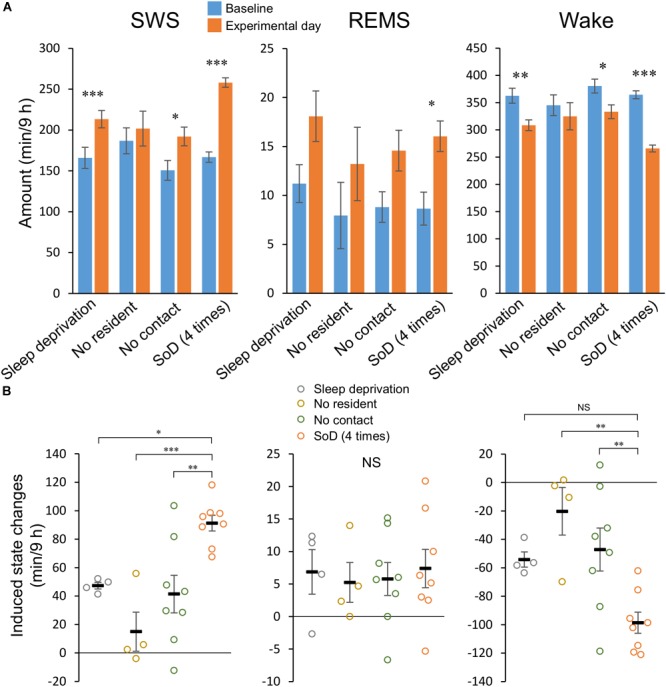
Social defeat (SoD) is essential for a strong slow-wave sleep (SWS) increase. **(A)** Total amounts of SWS, rapid eye movement sleep (REMS), and wakefulness for 9 h after a “Sleep deprivation” (*n* = 4), “No resident” (*n* = 4), “No contact” (*n* = 8), or SoD (*n* = 8) session with four defeats. ^∗^*P* < 0.05, ^∗∗^*P* < 0.01, and ^∗∗∗^*P* < 0.001 compared with baseline, assessed by paired two-tailed Student’s *t*-test. **(B)** Changes in the total amounts of SWS, REMS, and wakefulness for 9 h between baseline and experimental days. ^∗^*P* < 0.05, ^∗∗^*P* < 0.01, and ^∗∗∗^*P* < 0.001 compared with SoD, assessed by one-way ANOVA followed by Dunnett’s *post hoc* comparisons. Data are presented as means ± SEM. NS, not significant.

**FIGURE 4:**
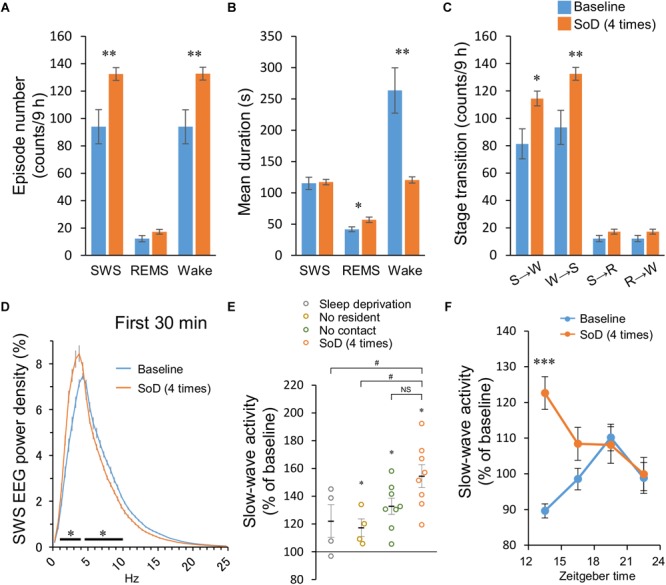
Sleep architecture after social defeat (SoD) stress. **(A–C)** Episode numbers **(A)**, mean episode durations **(B)**, and numbers of stage transitions **(C)** for 9 h after SoD session with four defeats. ^∗^*P* < 0.05 and ^∗∗^*P* < 0.01 compared with baseline, assessed by paired two-tailed Student’s *t*-test. **(D)** Power density of EEG during SWS for 30 min after SoD session with four defeats or onset of the dark phase on the baseline day. ^∗^*P* < 0.05 compared with baseline, assessed by two-way repeated measures ANOVA followed by Bonferroni’s *post hoc* comparisons. **(E)** Changes in the SWA (0.5–4 Hz) relative to the baseline for 30 min after a “Sleep deprivation,” “No resident,” “No contact,” or SoD session. ^∗^*P* < 0.05 compared with baseline, assessed by paired two-tailed Student’s *t*-test, #*P* < 0.05 compared with SoD, assessed by one-way ANOVA followed by Dunnett’s *post hoc* comparisons. **(F)** Time-course of SWA changes after SoD session. ^∗∗∗^*P* < 0.001 compared with baseline, assessed by two-way repeated measures ANOVA followed by Bonferroni’s *post hoc* comparisons.

Statistical analyses were carried out using GraphPad Prism software (GraphPad Software, La Jolla, CA, United States). All results are presented as mean ± standard error of the mean (SEM). A paired two-tailed Student’s *t*-test was used for statistical comparisons between paired groups (Figure 2B,C, 3A, 4A–C,E and Tables 2, 4). Two-way repeated-measures analysis of variance (ANOVA) followed by Bonferroni’s *post hoc* comparisons was used to analyze the sleep/wake profile (Figure 2A) and EEG power (Figure 4D,F). One-way ANOVA followed by Bonferroni’s (Figure 2D–F) or Dunnett’s (Figure 3B, 4E) *post hoc* comparisons were performed to compare groups of three or more. The Kruskal–Wallis test was used to analyze the data with significant variance heterogeneity assessed by Levene’s test (Figure 2D). In all cases, *P* < 0.05 was considered significant.

## Results

### SoD Stress Strongly Increases Sleep

We established a mouse model of sleep alterations after acute SoD stress based on a resident-intruder paradigm, whereby a C57BL/6j male mouse was introduced as an intruder to a resident CD-1 male mouse selected for its high level of aggression (Figure 1). To prevent injury to the intruder mouse, the intruder and resident mice were separated by a partition. During a 1-h SoD session, the partition was removed multiple times and reinserted every time when the intruder mouse was attacked and showed submissive behavior. After the SoD session, the intruder mouse was returned to the home cage and EEG and EMG of the intruder mice were recorded.

First, we investigated the extent to which sleep was affected by different numbers of defeats during a 1-h session. Intruder mice were exposed to two, four, or eight successive defeats. In each successive defeat, mice were in contact with the resident mice for 15.5 ± 1.5 s. We analyzed EEG and EMG recordings made after the SoD session or on the previous day (baseline). SWS strongly increased from the first 3 h after the sessions with two, four, and eight defeats. The strong increase in SWS lasted until 6 or 9 h after the sessions with two or four defeats, respectively. Accordingly, wakefulness was decreased during the corresponding periods. A REMS increase was observed only between 6 and 9 h after the SoD session with four successive defeats (Figure 2A and Table 1). When the first 3 h following the SoD sessions were analyzed in more detail, an increase in wakefulness only occurred during the first 10 min after the SoD sessions and SWS started to increase afterward (Figure 2B). Because most of the effects on sleep stages were observed within 9 h after the sessions, we compared the total amounts of 9 h following the SoD sessions (Figure 2C and Table 2). The total baseline amounts of the vigilance states during the same time period were not significantly different among groups [SWS: *P* = 0.4914, *F*(2,20) = 0.7364, REMS: *P* = 0.0761, *F*(2,20) = 2.938, Wake: *P* = 0.3578, *F*(2,20) = 1.083]. SWS significantly increased after SoD sessions, whereas wakefulness decreased accordingly. By contrast, REMS was only increased significantly after four defeats. Moreover, the calculated changes in the total amounts of SWS, REMS, and wakefulness between baseline and the post-SoD 9-h periods revealed a significant difference in REMS only between four and eight times (Figure 2D and Table 1). Because variance in SWS or wakefulness was highly significantly different among groups, we performed Bonferroni’s *post hoc* comparisons on the absolute deviations from the mean (Figure 2E). The deviations were significantly lower only in the intruder group with four defeats. Finally, we studied corticosterone responses in the intruder mice after SoD as an indicator of stress due to activation of the hypothalamic-pituitary-adrenal axis, the central stress response system (Mormède et al., 2007). Blood samples for measuring corticosterone levels were collected from undisturbed mice (to establish the baseline) and intruder mice immediately after the SoD sessions. A control session in a cage previously used by the resident mouse (“No resident”) was also conducted. SoD sessions significantly increased corticosterone levels and it was significantly stronger compared with “No resident” mice [*P* < 0.0001, *F*(4,26) = 24.5, one-way ANOVA followed by Bonferroni’s *post hoc* comparisons, Figure 2F]. These results may indicate that SoD causes stress in mice. However, there was no significant difference between SoD sessions with different numbers of successive defeats. These results suggested that the SWS increase observed after SoD stress is mostly independent of the numbers of defeats.

**Table 1:** Statistics of data shown in Figure 2A,B, 3B.

Two-way repeated measures ANOVA for Figure 2A			
		Main effect	Interaction
SoD (2 times)	SWS	*P* = 0.0057, *F*(1,49) = 8.359	*P* < 0.0001, *F*(6,49) = 6.405
*n* = 8	REMS	*P* = 0.9359, *F*(1,49) = 0.006532	*P* = 0.1535, *F*(6,49) = 1.65
	Wakefulness	*P* = 0.0124, *F*(1,49) = 6.744	*P* < 0.0001, *F*(6,49) = 5.89
SoD (4 times)	SWS	*P* < 0.0001, *F*(1,49) = 33.97	*P* < 0.0001, *F*(6,49) = 10.76
*n* = 8	REMS	*P* = 0.2284, *F*(1,49) = 1.488	*P* < 0.0001, *F*(6,49) = 8.931
	Wakefulness	*P* < 0.0001, *F*(1,49) = 29.37	*P* < 0.0001, *F*(6,49) = 11.06
SoD (8 times)	SWS	*P* < 0.0001, *F*(1,42) = 20.49	*P* = 0.0007, *F*(6,42) = 4.931
*n* = 7	REMS	*P* = 0.2984, *F*(1,42) = 1.109	*P* = 0.3936, *F*(6,42) = 1.074
	Wakefulness	*P* = 0.0005, *F*(1,42) = 14.38	*P* = 0.0049, *F*(6,42) = 3.698
**One-way ANOVA or Kruskal–Wallis test for Figure 2D, 3B**			
		**One-way ANOVA or Kruskal–Wallis test**	**Levene’s test**

Figure 2D	SWS	*P* = 0.5716, *H* = 1.118	*P* = 0.0004, *F*(2,20) = 11.69
	REMS	*P* = 0.0481, *F*(2,20) = 3.546	*P* = 0.9844, *F*(2,20) = 0.01575
	Wakefulness	*P* = 0.2035, *H* = 3.184	*P* = 0.0025, *F*(2,20) = 8.239
Figure 3B	SWS	*P* = 0.0006, *F*(3,20) = 8.986	*P* = 0.0838, *F*(3,20) = 2.56
	REMS	*P* = 0.9563, *F*(3,20) = 0.1049	*P* = 0.8879, *F*(3,20) = 0.2106
	Wakefulness	*P* = 0.0026, *F*(3,20) = 6.713	*P* = 0.1492, *F*(3,20) = 1.981

**Table 2:** Total amounts (minutes, mean ± SEM) of slow-wave sleep (SWS), rapid eye movement sleep (REMS), and wakefulness for 6, 9, 12, and 24 h after SoD sessions with two, four, or eight defeats.

		SoD (2 times) *n* = 8	SoD (4 times) *n* = 8	SoD (8 times) *n* = 7
**SWS (min)**			
6 h	Baseline	95.8 ± 8.9	97.2 ± 6.1	106.1 ± 14.5
	After session	153.9 ± 6.7***	152.4 ± 5.0***	154.9 ± 5.4**
9 h	Baseline	179.5 ± 11.4	166.8 ± 6.3	187.8 ± 17.9
	After session	249.6 ± 8.7**	258.0 ± 5.8***	255.2 ± 8.9**
12 h	Baseline	243.0 ± 12.6	221.5 ± 9.5	247.2 ± 18.3
	After session	317.8 ± 10.8**	315.3 ± 11.5***	325.1 ± 12.7***
24 h	Baseline	655.0 ± 14.9	638.8 ± 12.3	663.6 ± 16.5
	After session	712.3 ± 13.0**	725.3 ± 16.7***	735.5 ± 14.3**
**REMS (min)**
6 h	Baseline	4.0 ± 0.9	4.6 ± 1.0	7.3 ± 1.3
	After session	4.5 ± 1.3	4.3 ± 1.2	2.4 ± 0.8*
9 h	Baseline	10.7 ± 2.0	8.6 ± 1.7	16.0 ± 2.8
	After session	13.5 ± 3.0	16.0 ± 1.6*	12.0 ± 1.8
12 h	Baseline	14.3 ± 2.5	10.7 ± 1.8	21.1 ± 2.5
	After session	18.8 ± 3.6	20.9 ± 1.5**	16.9 ± 2.0
24 h	Baseline	70.9 ± 1.6	72.3 ± 3.8	77.3 ± 2.6
	After session	73.6 ± 3.9	79.9 ± 2.6	69.8 ± 3.7
**Wakefulness (min)**
6 h	Baseline	260.1 ± 9.3	258.3 ± 6.8	246.7 ± 15.4
	After session	201.5 ± 7.8***	203.3 ± 5.4***	202.7 ± 4.8*
9 h	Baseline	349.9 ± 12.0	364.6 ± 7.2	336.3 ± 20.0
	After session	276.9 ± 11.4**	265.9 ± 6.4***	272.7 ± 8.2**
12 h	Baseline	462.7 ± 14.0	487.8 ± 10.6	451.7 ± 20.1
	After session	383.4 ± 13.7**	383.9 ±12.0***	378.0 ± 11.7**
24 h	Baseline	714.1 ± 14.9	728.9 ± 13.7	699.0 ± 17.2
	After session	654.1 ± 16.1**	634.9 ± 18.0**	634.7 ± 11.3**

*^∗^*P* < 0.05, ^∗∗^*P* < 0.01, and ^∗∗∗^*P* < 0.001 compared with baseline, assessed by paired two-tailed Student’s *t*-test.*

### Specific Effects of SoD Versus Non-specific Effects of the SoD Procedure

To differentiate between the specific effects of SoD and non-specific effects of the SoD procedure (e.g., novelty or sleep deprivation), the sleep/wake behavior of mice after SoD stress was compared with that following other conditions, including sleep deprivation in a clean unused cage (“Sleep deprivation”), a cage previously used by the resident mouse (“No resident”), or the presence of the resident mouse separated by a partition (“No contact”). Data of the control conditions were compared with those of four successive defeats, because SWS consolidation occurred with significantly less variation after this SoD procedure than the ones with two or eight defeats.

During the “No resident” or “No contact” session, the animals were spontaneously active and awake during most of the 1-h session (Table 3). “Sleep deprivation” and “No contact” sessions significantly increased SWS and decreased wakefulness during a 9-h period after the sessions (Figure 3A and Table 4); however, the SWS increase was smaller than that after SoD stress (Figure 3B). SWS was not changed after the “No resident” session (Figure 3A). REMS also increased after the SoD and control procedures; however, there was only a significant difference between the baseline and experimental periods after SoD stress (Figure 3A). These results suggest that a significant part of the sleep changes after SoD can be attributed to non-specific sleep deprivation during the SoD session, i.e., about half of the increase of SWS and all of the increase of REM sleep may be a homeostatic sleep response to non-specific sleep deprivation.

**Table 3:** Percentage of animal behaviors (during wakefulness), slow-wave sleep (SWS), and rapid eye movement sleep (REMS) during the “No resident” and “No contact” sessions.

Behavior (%)	No resident	No contact
	*n* = 4	*n* = 8
Quiet waking	2.0 ± 0.8	3.9 ± 2.8
Grooming	14.5 ± 2.4	7.0 ± 1.5
Exploration	70.4 ± 6.9	79.2 ± 6.6
Consumption	3.1 ± 1.0	5.0 ± 0.8
SWS	9.6 ± 6.8	4.9 ± 4.5
REMS	0.3 ± 0.3	0.0 ± 0.0

**Table 4:** Total amounts (minutes, mean ± SEM) of slow-wave sleep (SWS), rapid eye movement sleep (REMS), and wakefulness for 6, 9, 12, and 24 h after “Sleep deprivation,” “No resident,” and “No contact” sessions.

		Sleep deprivation *n* = 4	No resident *n* = 4	No contact *n* = 8
**SWS (min)**
6 h	Baseline	94.4 ± 10.9	103.1 ± 9.9	93.7 ± 8.7
	After session	132.0 ± 12.8**	118.7 ± 18.5	119.9 ± 7.0**
9 h	Baseline	166.0 ± 12.9	186.7 ± 15.9	150.6 ± 12.2
	After session	213.3 ± 10.7***	201.7 ± 21.2	192.0 ± 11.5*
12 h	Baseline	228.3 ± 19.9	229.3 ± 18.0	214.4 ± 12.6
	After session	263.8 ± 23.8**	239.9 ± 17.3	242.8 ± 10.6
24 h	Baseline	619.6 ± 25.0	616.7 ± 8.4	623.1 ± 10.7
	After session	666.8 ± 37.3*	648.1 ± 13.7	653.9 ± 15.3
**REMS (min)**
6 h	Baseline	3.8 ± 1.3	3.7 ± 1.2	4.8 ± 1.0
	After session	8.6 ± 0.6	6.4 ± 1.9	7.0 ± 1.2
9 h	Baseline	11.2 ± 1.9	8.0 ± 3.4	8.8 ± 1.6
	After session	18.1 ± 2.6	13.2 ± 3.7	14.6 ± 2.1
12 h	Baseline	15.6 ± 2.1	9.4 ± 3.7	12.7 ± 1.9
	After session	21.6 ± 2.4	16.3 ± 3.3	17.8 ± 2.0
24 h	Baseline	77.2 ± 3.0	61.2 ± 2.2	70.6 ± 2.8
	After session	83.0 ± 3.3	74.7 ± 4.4	79.4 ± 3.9**
**Wakefulness (min)**
6 h	Baseline	261.8 ± 11.5	253.2 ± 11.0	261.6 ± 9.0
	After session	219.4 ± 12.4***	235.0 ± 20.3	233.1 ± 7.7**
9 h	Baseline	362.8 ± 13.7	345.3 ± 18.9	380.5 ± 12.8
	After session	308.7 ± 9.8**	325.1 ± 24.9	333.4 ± 12.7*
12 h	Baseline	476.1 ± 20.2	481.3 ± 21.4	492.9 ± 13.3
	After session	434.6 ± 22.7**	463.8 ± 20.1	459.4 ± 11.6
24 h	Baseline	743.2 ± 23.1	762.1 ± 6.3	746.3 ± 9.8
	After session	690.2 ± 39.8	717.2 ± 16.6	706.7 ± 15.7*

*^∗^*P* < 0.05, ^∗∗^*P* < 0.01, and ^∗∗∗^*P* < 0.001 compared with baseline, assessed by paired two-tailed Student’s*

### SoD Stress Increases the Number of SWS Episodes and Slow-Wave Activity

The sleep architecture and SWA were then analyzed after SoD session with four successive defeats. SoD stress significantly affected the episode number of SWS and wakefulness for 9 h after the SoD session (SWS: *P* = 0.0079, Wake: *P* = 0.0077, paired Student’s *t*-test, Figure 4A). The mean duration of wake episodes decreased by 48.4 ± 6.9% (*P* = 0.0035, paired Student’s *t*-test) compared with the baseline, whereas the duration of the SWS or REMS episodes was not significantly different or slightly increased, respectively (Figure 4B). The number of stage transitions from SWS to wake and wake to SWS was significantly increased (SWS to Wake: *P* = 0.0131, Wake to SWS: *P* = 0.0076, paired Student’s *t*-test), whereas other stage transitions were not affected by SoD stress (Figure 4C).

To assess whether EEG activity was altered by SoD stress, we compared the normalized EEG power spectrum of SWS for 30 min following the SoD session with baseline SWS. EEG activity was significantly increased in the frequency range of 1–4 Hz and decreased in the frequency range of 4.5–10 Hz during SWS [interaction *P* < 0.0001, *F*(49,350) = 25.47, two-way repeated-measures ANOVA followed by Bonferroni’s *post hoc* comparisons, Figure 4D]. SWA in the frequency range of 0.5–4 Hz was then calculated during the same period and compared with the “Sleep deprivation,” “No resident,” and “No contact” conditions. SWA was significantly increased in “No resident,” “No contact,” and SoD mice, although the increase of SWA in the SoD mice was significantly stronger than that in the “No resident” mice, but not in the “No contact” mice [*P* = 0.0176, *F*(3,20) = 4.258, one-way ANOVA followed by Dunnett’s *post hoc* comparisons, Figure 4E]. Finally, we calculated the 3-h SWAs in intruder mice for 12 h following SoD stress and found that the first 3-h SWA was significantly higher than the baseline SWA on the previous day [main effect *P* = 0.0005, *F*(1,28) = 15.66, interaction *P* = 0.0002, *F*(3,28) = 8.991, two-way repeated-measures ANOVA followed by Bonferroni’s *post hoc* comparisons, Figure 4F].

## Discussion

The present study explored the effect of acute SoD stress on the sleep/wake cycle in mice. We observed that SoD stress strongly promoted SWS over 9 h. Corticosterone, as an indicator of stress, was significantly increased in the blood plasma by SoD, confirming that SoD induced stress in the mice. The increase in SWS was associated with more transitions from wakefulness to SWS without changing the mean episode duration of SWS.

The effect of acute SoD stress on subsequent sleep amount was reported in several studies. One study showed strong SWS increase in mice after acute SoD stress by interaction for 1 h with mice from the same strain (Meerlo and Turek, 2001). By contrast, another study using the same procedure showed little effect of SoD on the SWS amount (Vaanholt et al., 2003). Due to the lack of information about the number and duration of attacks of the resident mice against the intruder mice, it is hard to explain the difference between the studies. Moreover, it was also reported that wakefulness is increased in the first 3 h after SoD followed by a sleep increase during later hours (Henderson et al., 2017). Highly aggressive male CD-1 mice were used in this study for the SoD of C57BL/6 mice during a 5-min interaction period followed by a 20-min period of olfactory, visual, and auditory contact between the resident and intruder mice. The initial increase of wakefulness in this study may be attributed to repeated painful attacks of the CD-1 mice against smaller C57BL/6 mice. Alternatively, the durations of physical and sensory stress (25 min) may be too short to induce sleep. Although variations in the aggressive behavior of the resident mice may account to some extent for the differences in the sleep/wake responses between the studies, sleep architecture, SWA, and corticosterone responses in socially defeated mice may also be influenced by other factors. For example, laboratory environment and time-of-the-day when the conflict was introduced may affect sleep and corticosterone parameters. Moreover, prior stress experiences of the intruder mice such as early life stress during maternal care, fighting between littermates, or low social ranking in the litter can also be important factors for outcomes after SoD stress (Wang et al., 2014; Peña et al., 2017). We designed a SoD stress protocol to minimize behavioral variations during SoD. In our protocol, intruder mice were separated from aggressive resident mice (CD-1 mice) when they exhibited submissive behaviors rather than by setting a fixed duration of interaction with the resident mice, preventing repeated and painful attacks against the submissive mouse. Moreover, physical interaction was conducted two, four or eight times to maintain a high level of SoD stress in the intruders during the session. We found that acute SoD stress, introduced at the end of the light period, increases SWS shortly after the SoD session. Moreover, the comparison of the specific effects of SoD and non-specific effects of the SoD procedure showed that a large portion of the sleep changes after SoD stress can be attributed to a homeostatic response to sleep deprivation. On the other hand, about half of the SWS increase is likely a specific response to SoD.

We also observed a strong increase in SWA after the SoD session (Figure 4E,F), consistent with previous reports in mice and rats (Meerlo et al., 1997; Meerlo and Turek, 2001; Kamphuis et al., 2015; Henderson et al., 2017). An increase in SWA is considered the hallmark of homeostatic sleep need in response to sleep deprivation (Suzuki et al., 2013; Dispersyn et al., 2017; Wang et al., 2018). The SWA increase, however, was stronger after the SoD condition than under sleep-disrupting control conditions without a CD-1 mouse. Moreover, SWS was still enhanced long after the SWA returned to the baseline level. These observations suggest that SWA is also affected by social stress and at least part of the SWS in response to SoD is independent of SWA.

Moreover, we observed large variations in the SWS increase after the SoD sessions, especially the ones with two and eight successive defeats (Figure 2D,E). It is well known that group-housed male mice establish a social hierarchy (Wang et al., 2014). As we did not control the social ranking of the intruder mice before the SoD session, it is possible that the SWS response of the intruder mice is affected by their SoD resilience.

The neuronal mechanisms of the sleep response to SoD stress are unknown. The mesolimbic dopamine system plays an important role in the development of depression-related behaviors after repeated SoD stress (Russo and Nestler, 2013). The mesolimbic system comprises dopaminergic projections from the ventral tegmental area to the nucleus accumbens, an area critical for reward and motivation (Graybiel, 2008; Russo and Nestler, 2013), and is also connected to the medial prefrontal cortex, amygdala, and hippocampus, allowing the system to integrate cognition, emotion, and action (Floresco, 2015). We and others recently described the role of the mesolimbic system in the gating of sleep by motivated behavior (Eban-Rothschild et al., 2016; Oishi et al., 2017a,b; Luo et al., 2018). Therefore, it is plausible that the mesolimbic system also mediates sleep responses to SoD stress.

Acute stress responses induce behavioral, sympathetic and neuroendocrine changes in animals to facilitate a fight or flight response (Chrousos, 2009). Sympathetic and neuroendocrine responses, including the increase of body temperature and heart rate and secretion of noradrenaline, adrenaline, and corticosteroids, usually dissipate within hours after acute SoD in rodents (Koolhaas et al., 1997, 2017). Sleep is known to inhibit glucocorticoid secretion in healthy men (Weitzman et al., 1983), whereas REMS disinhibition and dysregulation of hypothalamic-pituitary-adrenal axis are hallmarks of depression and chronic stress (Nollet et al., 2019; Steiger and Pawlowski, 2019). The increased SWS after SoD stress in our study may facilitate the termination of stress responses. Moreover, SWS induction may also be beneficial for restoration of brain homeostasis after the stress response (Xie et al., 2013; Ding et al., 2016). By contrast, REMS is considered to have an important function in the processing of emotional memory of adverse experiences to promote emotional and mental recovery (Suchecki et al., 2012; Goldstein and Walker, 2014). Only a moderate REMS increase was observed during a period 6–9 h after SoD sessions with four defeats (Figure 2A). Most, if not all, of the REMS increase, however, may be attributed to non-specific sleep deprivation during the SoD session and thus, a beneficial effect of REMS after SoD remains unclear.

In summary, our study revealed strong SWS responses to acute SoD stress in submissive male mice attempting to appease their aggressive counterpart, when painful attacks on the submissive mice were prevented. Our SoD stress model may be useful for studying the mechanisms and functions of sleep in response to social stress.

## Data Availability

All datasets generated for this study are included in the manuscript and/or the supplementary files.

## Author Contributions

SF and ML designed the experiments. SF, MKK, XZ, and MK collected and analyzed the data. SF and ML wrote the manuscript.

## Conflict of Interest Statement

The authors declare that the research was conducted in the absence of any commercial or financial relationships that could be construed as a potential conflict of interest.
